# Legal Notes for Institutional Workers

**Published:** 1919-05-03

**Authors:** 


### LEGAL NOTES FOR INSTITUTIONAL WORKERS.

### Unqualified Dentists.

The Dentists Act of 1878 (Sec. 3) provides that an unregistered person shall not "take or use" the name or title of "dentist" or of "dental practitioner," or any name, title, addition, or description implying that he is specially qualified to practise dentistry. In *Blain* v. *King*, 62 S. J. 520, a person described as an "artificial teeth specialist," but who was neither a registered dentist nor a qualified medical practitioner, stated in a County Court, while in the witness-box giving evidence, that he was a dentist. He was subsequently brought, on complaint, before the Justices, who convicted him of an offence under the statute. Against this decision he brought an appeal, which the King's Bench Division have, by a majority, dismissed. Mr. Justice Avory, with whom Mr. Justice Darling concurred, thought that it would be an undue limitation of the Dentists Act to say, as the appellant contended, that a person only committed an offence under it if he used the designation "dentist" with a view to obtaining practice or of advertising himself; the verbal announcement which the appellant made in this case was, in his opinion, a taking or using of the name and title of a dentist. Mr. Justice Shearman, however, took the view that the Act only struck at cases in which persons described themselves as dentists for the purpose of obtaining practice or attempting to obtain practice.

